# Traffic pollution and the incidence of cardiorespiratory outcomes in an adult cohort in London

**DOI:** 10.1136/oemed-2015-103531

**Published:** 2016-06-24

**Authors:** I M Carey, H R Anderson, R W Atkinson, S Beevers, D G Cook, D Dajnak, J Gulliver, F J Kelly

**Affiliations:** 1 Population Health Research Institute, St George's University of London, London, UK; 2 MRC-PHE Centre for Environment and Health, King's College London, London, UK; 3 UK Small Area Health Statistics Unit, MRC-PHE Centre for Environment and Health, Imperial College, London, UK

## Abstract

**Objectives:**

The epidemiological evidence for adverse health effects of long-term exposure to air and noise pollution from traffic is not coherent. Further, the relative roles of background versus near traffic pollution concentrations in this process are unclear. We investigated relationships between modelled concentrations of air and noise pollution from traffic and incident cardiorespiratory disease in London.

**Methods:**

Among 211 016 adults aged 40–79 years registered in 75 Greater London practices between 2005 and 2011, the first diagnosis for a range of cardiovascular and respiratory outcomes were identified from primary care and hospital records. Annual baseline concentrations for nitrogen oxide (NO_x_), particulate matter with a median aerodynamic diameter <2.5 μm (PM_2.5_) attributable to exhaust and non-exhaust sources, traffic intensity and noise were estimated at 20 m^2^ resolution from dispersion models, linked to clinical data via residential postcode. HRs were adjusted for confounders including smoking and area deprivation.

**Results:**

The largest observed associations were between traffic-related air pollution and heart failure (HR=1.10 for 20 μg/m^3^ change in NO_x_, 95% CI 1.01 to 1.21). However, no other outcomes were consistently associated with any of the pollution indicators, including noise. The greater variations in modelled air pollution from traffic between practices, versus within, hampered meaningful fine spatial scale analyses.

**Conclusions:**

The associations observed with heart failure may suggest exacerbatory effects rather than underlying chronic disease. However, the overall failure to observe wider associations with traffic pollution may reflect that exposure estimates based on residence inadequately represent the relevant pattern of personal exposure, and future studies must address this issue.

**What this paper adds**

- Epidemiological evidence for adverse health effects of long-term exposure to air and noise pollution from traffic is inconsistent.
- We investigated relationships between modelled concentrations of air and noise pollution from traffic and incident cardiorespiratory disease in London.
- The largest observed associations were between traffic-related air pollution and heart failure.
- No other outcomes were consistently associated with any of the pollution indicators, including noise.
- Future studies must address whether exposure estimates based on residence adequately represent the relevant pattern of personal exposure.

## Introduction

There is now an established body of epidemiological evidence linking long-term concentrations of air pollution to adverse health effects,^1^ in particular the risk of cardiovascular disease.^2^ Air pollution is believed to not only exacerbate existing heart conditions, but may also have a wider role in the development of the disease.^3^ While emissions from road traffic sources have been identified as a concern to public health,^4^ separating traffic emissions from the regional background pollution levels remains a continuing challenge,^5^ and ultimately it is still unclear whether primary traffic air pollution is, on a unit mass basis, more hazardous than background pollution.

Large-scale cohort studies have attempted to link different measures of road traffic exposure (related air pollutants, intensity or distance from road) to future disease development or mortality,^6–8^ but the overall body of evidence is not coherent. The European cohorts that comprised the ESCAPE (European Study of Cohorts for Air Pollution Effects) project did not find consistent associations between measures of traffic intensity and cardiovascular disease.^9^ Nor did the ESCAPE studies find consistent relationships when a range of elemental constituents of particles was considered instead as the exposure.^10^ On the other hand, there is growing evidence linking road traffic noise to an increased incidence of hypertension, myocardial infarction (MI) and stroke.^11^

Previously, we have used a national electronic database of primary care records to study the relationship between long-term exposure to air pollution and health.^12^ ^13^ The large-scale nature of these databases allow us to specifically address whether air pollution could have its effects by increasing the incidence of recorded disease. However, the scale of the pollution model (1 km^2^) previously limited our ability to investigate associations with the incidence of cardiovascular and respiratory disease arising from roadside traffic pollution. In this present study, we use modelled estimates for traffic pollutants and noise, and measures of traffic intensity at a finer spatial scale (residential postcode), to investigate relationships with disease incidence across Greater London.

## Methods

### Clinical data sources

The Clinical Practice Research Datalink (CPRD) is a large, validated primary care database that has been collecting anonymous patient data from participating UK general practices since 1987.^14^ It includes a full longitudinal medical record for all registered patients, which totalled over 12 million by the end of 2014. The database also contains a socioeconomic marker, the Index of Multiple Deprivation (IMD), a composite small-area (∼1500 people) measure used in England for allocation of resources.^15^ Approximately three-quarters of the contributing CPRD practices have consented to their data being linked to Hospital Episodes Statistics (HES) data, which record all clinical and administrative information on National Health Service (NHS)-funded inpatient episodes. Patient records are linked by a ‘trusted third party’ using their NHS number, sex, date of birth and postcode.

For this study, we selected practices within the study area bounded geographically by the orbital M25 motorway around Greater London. This identified 75 practices that were continually recording data between 2004 and 2011 within CPRD, and had given consent for their data to be linked to HES.

### Road traffic-based exposures

Three metrics of road traffic exposure were linked to the CPRD: (1) annual pollution concentrations, (2) traffic intensity or distance measures, (3) traffic noise levels.

Modelled annual concentrations for air pollutants were estimated using the KCLurban dispersion modelling system^16^ at a resolution of 20 m^2^. It incorporates hourly meteorological measurements, empirically derived concentrations of nitrogen oxides (NO_x_), ozone (O_3_) and particulate matter (PM), and information on source emissions from the London Atmospheric Emissions Inventory. Model validation was carried out by comparing observed versus modelled average monthly concentrations for each of the 96 months between 2003 and 2010. In this paper, we present two summaries of indicators of traffic pollution: NO_x_ and PM_2.5_ (mass of PM with a median aerodynamic diameter <2.5 μm) attributable to road traffic sources estimated from the sum of contributions from the following emissions sources: tyre, brake, exhaust, surface wear and resuspension. We also present results for the exhaust and non-exhaust road traffic PM_2.5_ components separately and for NO_2_ in supplementary analyses. Within London, the contribution of regional (background) PM_2.5_ to overall levels tends to dominate,^16^ and in our data, this contribution was >85% for 95% of our patients. Therefore, due to this lack of variation across London, we do not present any results for modelled total PM_2.5_.

Traffic proximity measures were developed relating to ‘heavy’ vehicle density, which was defined as: light goods vehicles, heavy goods vehicles (rigid and articulated trucks/lorries), buses and coaches. We included a distance measure (in metres) from the postcode centroid to the nearest road classified in the top quartile of heavy vehicle intensity. Traffic volume was estimated as total vehicle km driven (heavy vehicles only) in each year for all major roads that fall within a 100 m radius of the postcode address centroid. We used a cut-off >100 000 km driven to define ‘high volume’ in the analyses.

Road traffic noise levels were estimated using the TRAffic Noise EXposure (TRANEX) model.^17^ This uses information on road traffic flows and speeds, road geography, land cover, and building heights to estimate average sound-level pressure (L_Aeq_) in decibels (dB) over different time periods. Evaluations of TRANEX in other English cities have shown high correlation between modelled and measured 1-hour L_Aeq_ (Norwich: r=0.85, Leicester: r=0.95). In our analysis, we focused on average annual L_night_ recorded overnight between 23:00 and 07:00, as this period (1) represents when most of our study participants would be at their residence, (2) is when any effects of sleep deprivation are most likely. We provide alternative analyses using daytime noise (L_Aeq16_) in supplementary analyses, but since it was extremely highly correlated with night noise (r=0.998), it produced identical results. A sensitivity analysis was carried out that excluded patients in postcodes with significant non-traffic transport noise, defined as being within a 50 dB noise contour of Heathrow or City airport, or overland rail.

Finally for air pollution, the model estimates were interpolated to postcode level. In the UK, these were historically developed for national mail delivery, and are not necessarily geographically consistent units. They may contain up to 100 households, but will typically average about 15 households. We were able to map the address centroid for the 190 115 total London postcodes to the nearest centroid within each 20 m^2^ grid. For the noise model, the geometric centroids of the address locations in each postcode were directly used. These were then linked by a ‘trusted third party’ to CPRD, ensuring we had no direct access to the postcodes, preserving patient anonymity.

### Cohort and disease outcomes definition

In total, 223 264 adults were identified aged 40–79 years and registered on 1 January 2005 for >1 year continuously with their practice. From this group, 211 016 (95%) were successfully linked to our traffic-based exposures. Non-linkage was mainly due to a few practices being near the study area boundary, so many of their patients' individual postcodes were not eligible. A priori, we chose to assign each patient a fixed level of exposure based on the annual concentrations in the year before baseline (2004), mirroring our previous approach.^12^

The first occurrence on the general practitioner (GP) or hospital record from 1 January 2005 to 31 December 2011 of the following was searched for: coronary heart disease (CHD), MI, stroke, heart failure, hypertension, atrial fibrillation, chronic obstructive pulmonary disease (COPD) and pneumonia. Definitions used Read codes (GP record) based on the Quality and Outcomes Framework,^18^ which were mapped to corresponding International Classification of Diseases 10th revision (ICD)-10 codes, used on the hospital records. Detailed code listings are available from the authors. Patients with disease outcomes recorded at baseline were excluded from that particular analysis, while patients who de-registered from their practice were censored at that point in time.

Covariates for smoking and body mass index (BMI) were determined from the electronic record, using where possible the last recorded information prior to 2005. Some exceptions included (1) non-smokers who were reclassified as ex-smokers if they had older historical codes indicating smoking, (2) patients recorded as being a never or current smoker whose only recorded status was between 2005 and 2011, (3) patients with BMI values after 2005 that were closer in time to the baseline period.

### Statistical analyses

For each pollutant measure, we calculated intraclass correlation coefficients (ICCs) to estimate the proportion of total variance between the practice clusters. We used Cox proportional hazards models to investigate associations between all traffic exposure measures in the year before baseline (2004) and subsequent incidence. We adjusted cumulatively for (1) age, sex, smoking and BMI and (2) IMD decile. Alternate Cox models that stratified on these covariates made no appreciable difference (data not shown). We also investigated the impact of further adjusting pollutant measures for night-time noise and vice versa. To account for clustering by practice, the modified sandwich estimate of variance was used to produce robust SEs. We also investigated models which derive the contribution of between-practice and within-practice exposure to the overall effect.^12^ For air pollution concentrations, we summarised the hazard as approximate IQR changes (20 μg/m^3^ for NO_x_, 1 μg/m^3^ for PM_2.5_ and 0.3 μg/m^3^ for PM_2.5_ estimated from exhaust). For night-time noise we used a 5 dB change. All analyses were carried out in Stata V.13 (StataCorp LP, College Station, Texas, USA).

## Results

A summary of the 211 016 patients eligible for the analyses is shown in table 1. The cohort was 51% male with a mean age of 55.4 years. Table 2 describes the incidence of health outcomes during follow-up. For example, n=10 559 (5.00%) had a record of CHD and are not included in the denominator from which n=5925 (2.96%) were then identified as subsequently being diagnosed with CHD during 2005–2011. Deprivation was related to all incidence rates, except for atrial fibrillation, but was notably stronger for COPD and heart failure.

**Table 1:** Summary of cohort registered on 1 January 2005 (n=211 016)

Characteristic	Subgroup	N	Per cent
Gender	Men	107 226	50.8
	Women	103 790	49.2
Age	40–64	161 325	76.5
	65–79	49 691	23.6
Smoking†	Never	105 614	50.1
	Ex (amount unknown)	17 418	8.3
	Ex (light)	18 199	8.6
	Ex (heavy)	8840	4.2
	Current (amount unknown)	2294	1.1
	Current (light)	28 483	13.5
	Current (heavy)	14 503	6.9
	Missing	15 665	7.4
BMI	10–20	9589	4.5
	20–25	64 304	30.5
	25–30	61 384	29.1
	30–40	31 573	15.0
	40+	3436	1.6
	Missing	40 730	19.3
Index of Multiple Deprivation	1 (least deprived)	40 830	19.4
	2	49 494	23.5
	3	41 282	19.6
	4	51 123	24.2
	5 (most deprived)	28 287	13.4
Practice borough	Inner London	40 647	19.3
	Outer London	170 639	80.7
Time registered with practice	<10 years	79 797	37.8
	10+ years	131 219	62.2
High exposure to aircraft or rail noise*	No	155 670	73.8
	Aircraft or rail	55 346	26.2

*Lives in postcode within the 50 dB noise contour of Heathrow or City airport, or overland rail.

^†^Smoking amount categories were classed as light (1-19 cigarettes/day), heavy (20+ cigarettes/day) or unknown where not recorded.

BMI, body mass index.

**Table 2:** Disease outcomes summary between 2005 and 2011 (n=211 016)

		Has prior diagnosis on GP record by 1 January 2005		No history of disease on 1 January 2005	Receives first GP diagnosis or hospital admission 2005–2011		Most deprived IMD quintile vs least
Outcome	ICD-10 codes	n	Per cent	n	n	Per cent	Adjusted HR*
CHD	I20-I25	10 559	5.00	200 457	5925	2.96	1.19
Myocardial infarction	I21-I23	3974	1.88	207 042	2582	1.25	1.26
Stroke	I61, I63-I64	3969	1.88	207 047	3716	1.79	1.20
Heart failure	I50	1801	0.85	209 215	2224	1.06	1.80
Atrial fibrillation	I46-I49, R00.1	2967	1.41	208 049	4846	2.33	0.96
Hypertension	I10, I15	41 167	19.51	169 849	17 785	10.47	1.45
COPD	J41-J44	3780	1.79	207 236	7518	3.63	1.86
Pneumonia	J12-J18	3115	1.48	207 901	3587	1.73	1.50

*HR for IMD adjusted for age, sex, smoking and BMI.

BMI, body mass index; CHD, coronary heart disease; COPD, chronic obstructive pulmonary disease; GP, general practitioner; ICD, International Classification of Diseases; IMD, Index of Multiple Deprivation.

Table 3 summarises the markers of traffic pollution and noise used in the main analyses (with additional detail provided in online supplementary table S1). NO_x_ showed extremely high correlation (r=0.96) with PM_2.5_ attributable from traffic sources, but less so with night noise levels (r=0.40). The ICCs by practice were high for NO_x_ (ICC=0.80) and PM_2.5_ from traffic sources (ICC=0.67) demonstrating the majority of variation was between practice areas, whereas for night noise (ICC=0.05) most variation was within practice area. This contrast is visually demonstrated in online supplementary figure S1. Residents in areas with higher NO_x_ or PM_2.5_ tended to be younger by about 2.5 years on average. Approximately a fifth of the cohort was estimated to live within 100 m of a major road. Deprivation was related to all traffic measures, but strongest trends were seen with air pollution rather than distance or noise measures.

**Table 3:** Summary of NO_x_, PM_2.5_ (road traffic sources only), traffic volume, major road distance and night noise (n=211 016)

									Correlations				
Traffic exposure	Level	N	NO_x_ (mean ±SD)	PM_2.5_ due to traffic (mean ±SD)	L_night_ (mean ±SD)	Age at baseline (mean ±SD)	Current smokers (%)	Most deprived IMD 5th (%)	ICC*	NO_x_	PM_2.5_ traffic	Km driven	Distance to major road
NO_x_ (μg/m^3^)	0–55	68 644	48.2±4.4	1.0±0.1	50.9±2.9	56.4±11.2	21.7	6.4	0.80	–	0.96	0.47	−0.41
	55–75	101 287	63.3±5.4	1.4±0.2	51.8±4.1	55.4±11.0	20.6	12.7					
	75+	41 085	86.8±11.8	2.3±0.5	55.0±6.6	53.9±10.8	23.3	23.3					
PM_2.5_ from traffic sources (μg/m^3^)	0–1	32 322	44.8±3.5	0.9±0.1	50.1±1.8	56.4±11.2	22.2	6.1	0.67	0.96	–	0.58	−0.43
	1–2	152 042	62.0±9.1	1.4±0.3	51.6±3.8	55.5±11.1	20.8	11.2					
	2+	26 652	90.5±12.8	2.5±0.5	57.5±6.9	54.0±10.8	24.1	31.5					
Vehicle km driven†	None	119 984	58.8±11.6	1.3±0.3	49.9±1.2	55.5±11.0	20.8	11.1	0.07	0.47	0.58	–	−0.32
	Low volume	63 455	64.5±14.8	1.5±0.5	54.4±5.1	55.5±11.2	21.8	13.4					
	High volume	27 677	77.2±19.6	2.1±0.8	56.2±6.8	55.1±11.1	23.4	22.9					
Distance(m) to major road	>250 m	95 481	57.5±12.0	1.2±0.4	50.9±3.3	55.6±11.1	21.1	10.7	0.28	−0.41	−0.43	−0.32	–
	100–250 m	71 328	64.0±12.9	1.5±0.4	51.2±3.2	55.2±11.0	21.0	14.1					
	0–100 m	44 207	73.1±18.5	1.9±0.7	56.2±6.3	55.4±11.1	22.8	22.8					
L_night_ (dB)	0–55	172 940	60.8±13.2	1.4±0.4	50.1±1.2	55.4±11.1	21.3	13.1	0.05	0.40	0.52	0.59	−0.27
	55–60	16 467	64.8±15.6	1.6±0.5	57.5±1.5	55.9±11.1	21.1	11.3					
	60+	21 609	78.3±19.3	2.2±0.7	63.7±2.8	55.2±11.1	22.7	17.1					

*Intraclass correlation by practice cluster.

†Within 100 m radius of postcode address centroid only, with >100 000 km driven annually defined as high volume.

ICC, intraclass correlation coefficient; IMD, Index of Multiple Deprivation; NO_x_, nitrogen oxide; PM_2.5_, particulate matter with a median aerodynamic diameter <2.5 μm.

10.1136/oemed-2015-103531.supp2 Supplementary data

The results from the statistical models are shown in table 4 for CHD, MI, stroke and heart failure. There was little evidence that traffic pollution, intensity or noise was related to a higher incidence of CHD, MI or stroke, either before or after adjustment for deprivation. Only intensity and CHD showed a weak positive association after adjustment for deprivation. However, for heart failure, there was a positive association with NO_x_ and PM_2.5_ from traffic sources, which remained statistically significant after adjustment for area deprivation (eg, HR=1.10, 95% CI 1.01 to 1.21 for 20 μg/m^3^ NO_x_ increase). The relationship with distance measures and noise was also positive, but not statistically significant.

**Table 4:** HRs for incident CHD, MI, stroke and heart failure during 2005–2011 by traffic-related exposures

		CHD (n=200 457)		MI (n=207 042)		Stroke (n=207 047)		Heart failure (n=209 215)	
Exposure	Unit/category	HR1* (95% CI)	HR2† (95% CI)	HR1* (95% CI)	HR2† (95% CI)	HR1* (95% CI)	HR2† (95% CI)	HR1* (95% CI)	HR2† (95% CI)
NO_x_	20 μg/m^3^ change	1.00 (0.96 to 1.04)	0.97 (0.93 to 1.01)	0.94 (0.88 to 1.01)	0.91 (0.84 to 0.98)	0.94 (0.89 to 0.99)	0.90 (0.85 to 0.96)	1.18 (1.08 to 1.28)	1.10 (1.01 to 1.21)
Per cent of PM_2.5_ due to traffic	1 μg/m^3^ change	0.99 (0.94 to 1.05)	0.96 (0.91 to 1.02)	0.92 (0.84 to 1.02)	0.88 (0.79 to 0.98)	0.93 (0.86 to 1.10)	0.88 (0.81 to 0.97)	1.25 (1.11 to 1.40)	1.15 (1.02 to 1.30)
Vehicle km driven‡	None	1	1	1	1	1	1	1	1
	Low volume	0.95 (0.91 to 1.01)	0.95 (0.90 to 1.00)	0.99 (0.91 to 1.08)	0.98 (0.90 to 1.07)	1.02 (0.96 to 1.11)	1.02 (0.95 to 1.10)	0.98 (0.90 to 1.06)	0.96 (0.88 to 1.04)
	High volume	1.07 (1.00 to 1.15)	1.05 (0.98 to 1.13)	0.99 (0.88 to 1.12)	0.97 (0.86 to 1.09)	1.00 (0.88 to 1.15)	0.98 (0.86 to 1.12)	1.09 (0.96 to 1.23)	1.02 (0.90 to 1.16)
Distance (m) to major road	>250 m	1	1	1	1	1	1	1	1
	100 to 250 m	1.00 (0.94 to 1.07)	0.99 (0.93 to 1.06)	0.96 (0.88 to 1.06)	0.95 (0.87 to 1.05)	1.01 (0.94 to 1.09)	1.00 (0.93 to 1.08)	1.09 (1.00 to 1.20)	1.06 (0.97 to 1.17)
	0 to 100 m	1.03 (0.96 to 1.11)	1.02 (0.95 to 1.09)	0.97 (0.87 to 1.09)	0.96 (0.85 to 1.07)	0.99 (0.89 to 1.11)	0.98 (0.87 to 1.09)	1.06 (0.95 to 1.19)	1.02 (0.91 to 1.14)
L_night_	<55 dB	1	1	1	1	1	1	1	1
	55 to 60	0.88 (0.79 to 0.99)	0.88 (0.79 to 0.98)	0.90 (0.79 to 1.03)	0.91 (0.79 to 1.03)	0.97 (0.86 to 1.09)	0.97 (0.86 to 1.10)	0.87 (0.74 to 1.02)	0.88 (0.75 to 1.03)
	60–	1.01 (0.93 to 1.10)	1.00 (0.93 to 1.09)	1.00 (0.86 to 1.15)	0.99 (0.86 to 1.14)	0.93 (0.83 to 1.05)	0.92 (0.82 to 1.04)	1.12 (0.96 to 1.29)	1.09 (0.94 to 1.26)

*HR1: age, gender, smoking and BMI.

†HR2: as HR1, plus additional adjustment for IMD.

‡Within 100 m radius of postcode address centroid only, with >100 000 km driven annually defined as high volume.

BMI, body mass index; CHD, coronary heart disease; IMD, Index of Multiple Deprivation; MI, myocardial infarction; NO_x_, nitrogen oxide; PM_2.5_, particulate matter with a median aerodynamic diameter <2.5 μm.

Table 5 summarises results for hypertension, atrial fibrillation, COPD and pneumonia. While all outcomes showed positive associations with air pollution, these were generally explained by adjustment for area deprivation. For intensity and distance measures, the strongest trends were seen with pneumonia and distance from major road (HR=1.06, 95% CI 0.98 to 1.14 for 0 to 100 vs >250 m). There was no evidence of an association between hypertension and night noise (HR=0.99, 95% CI 0.94 to 1.05 for 60+ vs <55 dB), which remained true when analyses were restricted to patients resident in areas not subject to high levels of aircraft or rail noise (see online supplementary table S2).

**Table 5:** HRs for incident hypertension, atrial fibrillation, COPD and pneumonia during 2005–2011 by traffic-related exposures

		Hypertension (n=169 849)		Atrial fibrillation (n=208 049)		COPD (n=207 236)		Pneumonia (n=207 901)	
Exposure	Unit/category	HR1* (95% CI)	HR2† (95% CI)	HR1* (95% CI)	HR2† (95% CI)	HR1* (95% CI)	HR2† (95% CI)	HR1* (95% CI)	HR2† (95% CI)
NO_x_	20 μg/m^3^ change	1.10 (1.01 to 1.21)	1.05 (0.97 to 1.14)	1.10 (1.01 to 1.21)	1.05 (0.97 to 1.14)	1.06 (0.90 to 1.25)	0.99 (0.86 to 1.13)	1.11 (1.02 to 1.19)	1.06 (0.98 to 1.14)
Per cent of PM_2.5_ due to traffic	1 μg/m^3^ change	1.11 (0.97 to 1.26)	1.03 (0.91 to 1.16)	0.97 (0.90 to 1.04)	0.98 (0.91 to 1.06)	1.08 (0.86 to 1.34)	0.98 (0.81 to 1.18)	1.11 (1.00 to 1.23)	1.04 (0.95 to 1.15)
Vehicle km driven‡	None	1	1	1	1	1	1	1	1
	Low volume	0.99 (0.93 to 1.06)	0.98 (0.93 to 1.04)	0.99 (0.93 to 1.06)	0.98 (0.93 to 1.04)	0.97 (0.89 to 1.06)	0.96 (0.89 to 1.03)	1.05 (0.97 to 1.14)	1.03 (0.95 to 1.12)
	High volume	1.05 (0.97 to 1.13)	0.99 (0.93 to 1.06)	1.05 (0.97 to 1.13)	0.99 (0.93 to 1.06)	1.01 (0.91 to 1.11)	0.94 (0.86 to 1.03)	1.05 (0.96 to 1.16)	1.01 (0.91 to 1.11)
Distance(m) to major road	>250 m	1	1	1	1	1	1	1	1
	100 to 250 m	0.99 (0.93 to 1.06)	0.98 (0.93 to 1.04)	1.01 (0.95 to 1.06)	1.00 (0.95 to 1.06)	0.94 (0.86 to 1.03)	0.92 (0.84 to 1.01)	1.05 (0.97 to 1.13)	1.03 (0.96 to 1.11)
	0 to 100 m	1.05 (0.97 to 1.13)	0.99 (0.93 to 1.06)	1.02 (0.94 to 1.10)	1.02 (0.94 to 1.11)	0.93 (0.84 to 1.02)	0.90 (0.81 to 0.99)	1.08 (1.01 to 1.17)	1.06 (0.98 to 1.14)
L_night_	<55 dB	1	1	1	1	1	1	1	1
	55 to 60	1.00 (0.93 to 1.07)	1.01 (0.94 to 1.08)	0.93 (0.85 to 1.02)	0.93 (0.85 to 1.01)	0.87 (0.77 to 0.99)	0.88 (0.77 to 1.00)	0.95 (0.84 to 1.07)	0.95 (0.85 to 1.08)
	60–	1.01 (0.95 to 1.07)	0.99 (0.94 to 1.05)	0.95 (0.88 to 1.03)	0.95 (0.88 to 1.04)	1.00 (0.91 to 1.10)	0.98 (0.89 to 1.08)	0.98 (0.88 to 1.08)	0.96 (0.87 to 1.07)

*HR1: age, gender, smoking and BMI.

†HR2: as HR1, plus additional adjustment for IMD.

‡Within 100 m radius of postcode address centroid only, with >100 000 km driven annually defined as high volume.

BMI, body mass index; COPD, chronic obstructive pulmonary disease; IMD, Index of Multiple Deprivation; NO_x_, nitrogen oxide; PM_2.5_, particulate matter with a median aerodynamic diameter <2.5 μm.

Associations with all traffic-related outcomes were similar when the cohort was restricted to patients registered for >10 years with their practice (see online supplementary table S2). Model effect estimates were broadly similar when they fitted separately to younger and older participants (see online supplementary table S3), or to never-smokers and current smokers (see online supplementary table S4).

Measures of traffic pollution (NO_2_ and PM_2.5_ traffic exhaust and non-exhaust) produced identical findings due to the high correlation between them (see online supplementary table S5). Partitioning the overall effect into between-practice and within-practice estimates for traffic air pollution and noise (see online supplementary table S6) tended to suggest stronger positive effects between practices for all outcomes except for MI, but precision was wide. Further adjusting the air pollution associations for night noise, or vice versa, made no material difference (data not shown).

## Discussion

In a longitudinal study using linked electronic primary care and hospital admission records in Greater London, we investigated the associations between cardiorespiratory outcomes and three indicators of exposure to traffic pollution: modelled air pollutants, modelled noise and traffic proximity. Overall, associations between the health outcomes and the various indicators of exposure to traffic pollution were small, inconsistent and lacking in precision but some trends with heart failure and pneumonia were observed.

### Strengths and limitations

We have previously used similar methodology to study disease incidence in the 2000s across England nationally in CPRD,^12^ which was similarly inconclusive. While we argued the benefits of linked primary care databases in carrying out large epidemiological analyses, we noted a possible limitation that the earlier pollution model's resolution (1 km^2^) could not account for potential within urban variations, due to busy roads for example. The improved resolution of the dispersion model in this study (20 m^2^), which can estimate significant changes in exposure of air pollution (NO_x_, NO_2_) between major roads and suburban background locations,^16^ offers a potential benefit to directly study the effects of traffic pollution.

Once modelled air pollution data were linked to patients residential postcodes however, subtle roadside changes predicted by the model were small in comparison to the larger differences estimated between areas, even within Greater London. This is demonstrated by large ICCs (>0.65) for both air pollutants, revealing most modelled variation was between (practice) areas. In other words, patients in the top 10% of NO_x_ exposure in our study, for example, were far more likely (77%) to be from an Inner London practice than those not in the top 10% of NO_x_ (13%). The statistical implication is that the models are predominately estimating a between (practice) area effect for air pollutants, which was confirmed when we partitioned the overall estimate into between-practice and within-practice effects. The addition of distance and intensity measures in our study provided a less problematic approach, but did not produce any further evidence of associations with traffic. Another statistical issue was the strong correlation (>r=0.95) between NO_x_ and our traffic components of PM_2.5_, which effectively eliminated our ability to discern between different contributors of emissions (exhaust vs non-exhaust) or mutually adjust for them. While high correlation between different measures of traffic pollution is to be expected, it may be the dispersion model is being too closely driven by the same predictors, and is underestimating the variation which may be expected from actual measurements.

The lack of variation in our modelled air pollution estimates could be a result of: (1) our sample of practices being under-represented by areas where patients live by busy roads, (2) a high proportion of addresses near busy roads being mapped to postcode centroids which lie further away from the road or (3) in outer boroughs of London, a much smaller proportion of residents live in close proximity to busy roads. By contrast, road traffic noise varied far more within each area, suggesting that there were patients in *all* practices with exposure to high levels of road traffic noise. People from different areas even within the same city, will differ for many reasons besides air quality such as lifestyle or ethnicity, and although adjusting for area deprivation partially addresses this, we cannot discount residual confounding in our results. Finally, like most other large-scale cohorts of long-term exposure to air pollution, we acknowledge that modelled exposure, however accurate, will only ever be a proxy for real long-term or even lifetime exposure. This is further complicated in London by: (1) a large proportion who commute to work on public transport travelling outside their residential area, (2) a ‘revolving door’ population where it is estimated every year around 9% of its population moves into London while almost 7% leaves its territory.^19^ However, sensitivity analyses restricting to patients who had been registered with their practice for >10 years did not alter our findings.

Finally, a further weakness of our study was the lack of more individual-level confounders in the analysis such as ethnicity and educational status, although these will be partly accounted for in the IMD. However, we do not believe that the absence of these would account for the overall lack of associations we found across the different exposures and outcomes.

### Recent literature of traffic pollution and health in cohort studies

While the effects of long-term exposure to ambient air pollution have been studied in many worldwide settings, few cohort studies have focused on primary traffic pollutants such as NO_x_ or NO_2_, and of those that have, have used mortality as the outcome. The Dutch netherlands cohort study on diet and cancer (NLCS) study^6^ found associations between NO_2_ and black smoke and mortality, with associations highest for respiratory causes. Recent studies in Rome^8^ and California^7^ also reported positive associations between NO_2_ and mortality, with strongest trends among deaths from cardiovascular causes. While the ESCAPE meta-analyses of 11 European cohorts^20^ found associations between PM_10_, PM_2.5_ and the incidence of coronary events, these relationships were not seen for either NO_x_ or NO_2_. Another ESCAPE meta-analyses of 19 cohorts was unable to find consistent evidence between a comprehensive set of elemental constituents of PM and overall cardiovascular mortality.^10^

Recent cohort studies have measured alternative measures of traffic pollution, such as intensity on the nearest road^6^ ^8^ or road traffic noise.^21^ The NLCS study found elevated associations between traffic intensity and mortality from ischaemic heart disease (IHD), cerebrovascular causes and heart failure, not explained by adjustment for traffic noise. Evidence from ESCAPE also showed associations between traffic load on major roads within 100 m of residence and hypertension across 15 population-based cohorts.^22^ Meta-analyses of road traffic noise mainly across Europe showed a 3% increased risk in hypertension prevalence per 5 dB increase in daytime noise,^23^ and a 8% increase in CHD risk per 10 dB of weighted day-noise level.^24^

### How our study fits in

While we were unable to replicate many of the positive findings from recent cohort studies, there are important differences to consider. In the Rome study,^8^ associations with their indicators of traffic were only statistically significant after adjustment for socioeconomic status. Adjustment for deprivation in our study had the opposite effect, as more deprived areas were associated with more traffic pollution in our sample of practices in Greater London. This was a pattern we previously observed nationally,^12^ and has been recently replicated by a study of air pollution inequality at regional and city levels across England.^25^ Studies which additionally explored individual as well as neighbourhood measures of socioeconomic status, generally replicated this relationship,^26^ with New York a notable exception where affluent areas were located in high-density areas close to busy roads.^1^ Increased gentrification of inner cities over time may change the relationship between pollution and socioeconomic status, and although we found no evidence of this, we were likely under-represented in very central affluent London areas. However, this seems an unlikely explanation for the lack of associations with air pollution that were mostly null before any adjustments for deprivation. Another explanation may be the reduced exposure range in comparison to previous studies such as the American Cancer Society,^1^ where our IQR for modelled PM_2.5_ in Greater London in 2004 (1 μg/m^3^) was approximately a quarter of what was estimated in the ACS in 1999–2000. However, many of our HRs were very close to or below 1, suggesting further scaling of estimates would still not produce comparable associations.

We also found little evidence of any associations with traffic noise, whether we used daytime or night noise measures which were very highly correlated. This contrasts with recent findings linking noise levels derived from the same model to hospital admissions for stroke across London.^27^ In addition, it had little effect when added as an adjustment factor when estimating associations with air pollution. The failure to find any association with hypertension contrasts with a predominately European meta-analysis of 24 observational studies from 1970 to 2010, all smaller in size to ours.^23^ It may be that the traffic noise model used here is too crude to detect small health effects, failing to account for location of bedrooms within a house, or whether windows are open or closed at night.^11^ Aircraft noise levels in London have been shown to be associated with increased risks of stroke and CHD hospital admissions and mortality.^28^ However, excluding patients who lived in areas exposed to major levels of noise pollution from aircraft or rail, did not materially alter our findings for traffic noise. Finally, the exposure range in noise levels across greater London may be different to studies that have shown positive associations. For example, the IQR of estimated night noise levels in Vancouver (50–58 dB)^29^ was twice that seen in our study(49–52 dB). The NLCS study^21^ used a reference category of ≤50 dB daytime noise in analyses; by contrast, in our study, nobody was estimated to have daytime noise of ≤54 dB. The Vancouver study also suggested that the relationship between noise and CHD mortality was non-linear and only seen in the top decile;^29^ however, risks were similar when we compared patients in the top decile of exposure (≥60 dB) to those with lower categories.

### Heart failure and pneumonia

Our analyses provided evidence linking exposure to air pollution from traffic and the incidence of heart failure and to lesser extent pneumonia, which follows on from similar associations found with air pollution in our national study on incidence^12^ and mortality.^30^ Neither disease outcome has been well studied among the air pollution literature, though a link between air pollution and heart failure has been recently speculated on,^3^ and a meta-analysis of time-series studies estimated increased risk of hospitalisation or death from heart failure with daily levels of PM_2.5_ and NO_2._^31^ The NLCS study^21^ reported associations with heart failure mortality and pollution concentrations at home address (NO_2_, PM_2.5_), but not with intensity or distance from major road, which were unaffected by adjustment for noise, all which mirrored our findings. Most air pollution studies of pneumonia have focused on short-term effects of exposure, but a study in Canada found associations with long-term exposure to NO_2_ and PM_2.5_ and hospitalisation for community-acquired pneumonia.^32^

As heart failure often represents the end stage for cardiovascular disease, associations here may represent an exacerbatory effect of air pollution in a group primarily older with more comorbidity.^33^ For example, among our heart failure incident cases two-thirds already had been diagnosed with COPD at baseline, while about a third (31%) had CHD; two-thirds (68%) of pneumonia cases were aged ≥60 years. There is a strong socioeconomic trend with heart failure,^34^ also seen in our study, which suggests we cannot rule out residual confounding as an explanation as we were unable to adjust for individual deprivation.

## Conclusion

Our results suggest that adults living in inner London, or near busy roads, are not at greater risk of developing cardiorespiratory diseases despite being potentially exposed to higher average levels of traffic pollution and noise. They may, however, be at increased risk of exacerbations of heart failure and pneumonia which are more likely to result from shorter term exposure. We cannot rule out associations with longer term exposure and underlying disease, as our pollution models cannot accurately represent the reality of long-term exposure for individuals, especially within a dynamic population such as London. Although our large cohort study offers greater statistical power, future smaller studies with better exposure assessment may be of more value. Only by shifting measurement of exposure from places to people will we be better able to answer the epidemiological question of whether traffic pollution leads to more disease.
