# Supplementary material for: Traffic pollution and the incidence of cardiorespiratory outcomes in an adult cohort in London
Source: Occup Environ Med. 2016 Jun 24;73(12):849–56. doi: 10.1136/oemed-2015-103531 (PMC5241502; doi:10.1136/oemed-2015-103531)
Supplement: Supplementary data [file oemed-2015-103531supp.pdf]

Supplemental Material

Title: Traffic pollution and the incidence of cardio-respiratory outcomes in an adult cohort in London

Authors: IM Carey, HR Anderson, RW Atkinson, S Beevers, DG Cook, D Dajnak, J Gulliver, FJ Kelly.

Contents

- **Figure S1** – Practice means, and 10th and 90th percentiles for NO_x and L_{night}
- **Table S1 –** Mean and correlation summary for traffic air pollutants and night noise
- **Table S2** – Hazard ratios for incident CHD, heart failure, hypertension during 2005-11 by NO_x, $PM_{2.5}$ (traffic sources only) and L_{night} restricted to patients based on registration length or aircraft/rail noise profiles
- **Table S3** - Hazard ratios for all outcomes during 2005-2011 for NOx, PM2.5 (traffic sources only) and L_{night} stratified by age
- **Table S4** - Hazard ratios for all outcomes during 2005-2011 for NOx, PM2.5 (traffic sources only) and L_{night} stratified by smoking
- **Table S5 –** Hazard ratios for all outcomes during 2005-2011 for pollutants not in main paper - NO_2, $PM_{2.5}$ (exhaust) and $PM_{2.5}$ (traffic non-exhaust)
- **Table S6** – Hazard ratios for between and within practice effects for all outcomes during 2005-2011 by NO_x, $PM_{2.5}$ (traffic sources only) and L_{night}

Figure S1 – Practice means, and 10th and 90th percentiles for NO_x and L_{night}

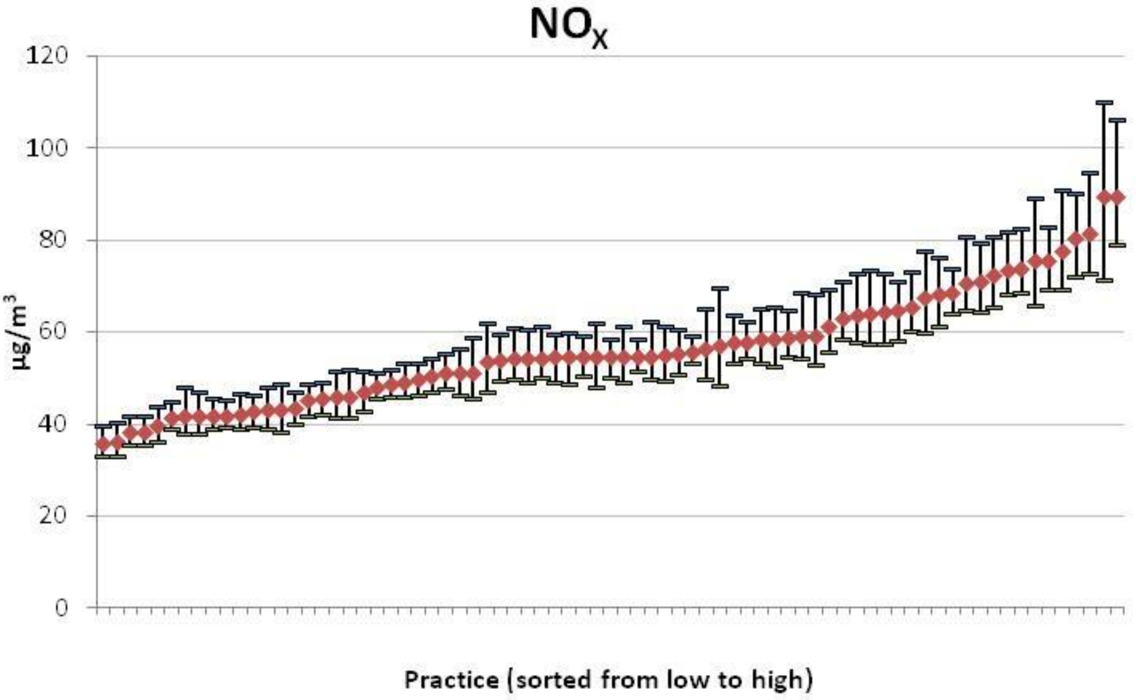

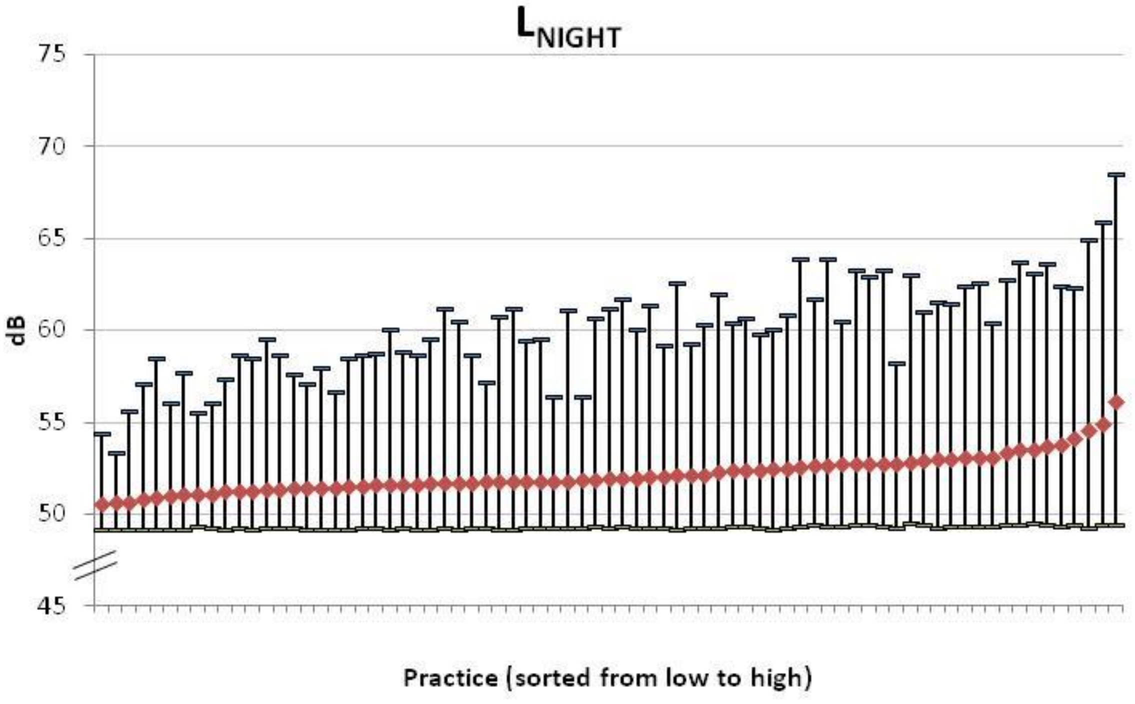

Table S1 – Mean and correlation summary for traffic air pollutants and night noise

Traffic Exposure	Mean	Std Dev	Inter Quartile Range	ICC†	Correlations between pollutants					
					NO_2	NO_X	$PM_{2.5}$ exhaust	$PM_{2.5}$ non-exhaust	$PM_{2.5}$ traffic sources	L_{night}
**NO_2**	37.4	5.8	7.6	0.86	_	0.99	0.93	0.92	0.94	0.35
**NO_X**	63.0	15.1	18.6	0.80		_	0.95	0.94	0.96	0.40
**$PM_{2.5}$ exhaust**	0.80	0.30	0.31	0.70			_	0.95	0.99	0.49
**$PM_{2.5}$ non-exhaust**	0.65	0.23	0.28	0.61				_	0.99	0.56
**Combined $PM_{2.5}$ from traffic sources**	1.45	0.52	0.60	0.67					_	0.52
**Night Road Traffic Noise (L_{night})**	52.1	4.6	2.6	0.05						_

Table S2 – Hazard ratios for incident CHD, heart failure, hypertension during 2005-11 by NO_x, $PM_{2.5}$ (traffic sources only) and L_{night} restricted to patients based on registration length or aircraft/rail noise profiles

**Traffic Exposure**	Unit/category	**CHD**				**Heart failure**				**Hypertension**			
		Base model		Base + IMD		Base model		Base + IMD		Base model		Base + IMD	
		**HR**	95% CI	**HR**	95% CI	**HR**	95% CI	**HR**	95% CI	**HR**	95% CI	**HR**	95% CI
Patients registered in same practice for 10+ yrs (n=131,219)													
**NO_X**	20μg/m³ change	**0.99**	0.93-1.04	**0.96**	0.91-1.01	**1.18**	1.07-1.30	**1.11**	1.01-1.23	**1.08**	1.00-1.17	**1.04**	0.96-1.13
**$PM_{2.5}$ Traffic**	1μg/m³ change	**0.98**	0.91-1.05	**0.94**	0.87-1.02	**1.25**	1.10-1.42	**1.16**	1.02-1.32	**1.08**	0.96-1.21	**1.02**	0.90-1.14
**L_{night}**	60-dB vs. <55dB	**0.98**	0.87-1.10	**0.97**	0.87-1.09	**1.02**	0.84-1.24	**1.00**	0.82-1.22	**0.99**	0.92-1.06	**0.97**	0.91-1.04
Patients resident in area of non Aircraft or Rail noise pollution (n=155,670)													
**NO_X**	20μg/m³ change	**1.00**	0.96-1.04	**0.97**	0.93-1.02	**1.22**	1.11-1.33	**1.15**	1.03-1.28	**1.11**	1.01-1.23	**1.06**	0.97-1.16
**$PM_{2.5}$ Traffic**	1μg/m³ change	**1.00**	0.94-1.06	**0.97**	0.91-1.03	**1.30**	1.15-1.46	**1.21**	1.05-1.38	**1.13**	0.98-1.29	**1.05**	0.92-1.20
**L_{night}**	60-dB vs. <55dB	**0.99**	0.90-1.09	**0.99**	0.90-1.08	**1.12**	0.95-1.32	**1.10**	0.93-1.29	**1.01**	0.95-1.08	**1.00**	0.94-1.06

Base model: Age, gender, smoking & BMI.

Table S3 – Hazard ratios for all outcomes during 2005-2011 for NO_x, $PM_{2.5}$ (traffic sources only) and L_{night} stratified by age

Exposure	Age (yrs)	Unit	CHD		MI		Stroke		Heart failure	
			HR	95% CI	HR	95% CI	HR	95% CI	HR	95% CI
**NO_X**	Age <65y	20μg/m³ change	**0.95**	0.90-1.01	**0.87**	0.78-0.96	**0.86**	0.79-0.92	**1.04**	0.89-1.23
	Age ≥65y	20μg/m³ change	**1.00**	0.93-1.06	**0.94**	0.86-1.04	**0.94**	0.86-1.02	**1.13**	1.03-1.24
**$PM_{2.5}$ Traffic**	Age <65y	1μg/m³ change	**0.95**	0.88-1.03	**0.86**	0.75-0.98	**0.82**	0.73-0.92	**1.06**	0.84-1.33
	Age ≥65y	1μg/m³ change	**0.97**	0.88-1.07	**0.90**	0.78-1.04	**0.94**	0.83-1.06	**1.20**	1.07-1.34
**L_{night}**	Age <65y	60-dB vs. <55dB	**1.01**	0.90-1.13	**1.03**	0.86-1.24	**0.88**	0.78-1.17	**0.99**	0.75-1.32
	Age ≥65y	60-dB vs. <55dB	**1.00**	0.88-1.13	**0.95**	0.78-1.16	**0.95**	0.82-1.10	**1.12**	0.96-1.31
			**Hypertension**		**Atrial fibrillation**		**COPD**		**Pneumonia**	
**NO_X**	Age <65y	20μg/m³ change	**1.03**	0.95-1.13	**0.94**	0.87-1.01	**1.00**	0.85-1.17	**1.03**	0.94-1.13
	Age ≥65y	20μg/m³ change	**1.10**	1.01-1.19	**1.01**	0.95-1.07	**0.98**	0.86-1.11	**1.08**	1.00-1.17
**$PM_{2.5}$ Traffic**	Age <65y	1μg/m³ change	**1.01**	0.89-1.14	**0.93**	0.83-1.04	**1.01**	0.82-1.25	**1.01**	0.89-1.15
	Age ≥65y	1μg/m³ change	**1.10**	0.98-1.24	**1.02**	0.93-1.11	**0.95**	0.80-1.13	**1.08**	0.98-1.20
**L_{night}**	Age <65y	60-dB vs. <55dB	**0.95**	0.89-1.01	**0.92**	0.78-1.09	**1.03**	0.89-1.19	**0.97**	0.81-1.15
	Age ≥65y	60-dB vs. <55dB	**1.09**	1.01-1.19	**0.97**	0.88-1.07	**0.93**	0.81-1.07	**0.96**	0.84-1.11

All Hazard Ratios adjusted for Age, gender, smoking, BMI & IMD.

Table S4 – Hazard ratios for all outcomes during 2005-2011 for NO_x, $PM_{2.5}$ (traffic sources only) and L_{night} stratified by smoking

Exposure	Smoking	Unit	CHD		MI		Stroke		Heart failure	
			HR	95% CI	HR	95% CI	HR	95% CI	HR	95% CI
**NO_X**	Never	20µg/m³ change	**1.02**	0.95-1.09	**0.92**	0.82-1.03	**0.87**	0.80-0.94	**1.11**	0.99-1.25
	Current	20µg/m³ change	**0.94**	0.88-0.99	**0.89**	0.81-0.97	**0.90**	0.80-1.01	**1.17**	1.03-1.34
**$PM_{2.5}$ Traffic**	Never	1µg/m³ change	**1.03**	0.93-1.14	**0.90**	0.77-1.06	**0.84**	0.74-0.96	**1.14**	0.98-1.32
	Current	1µg/m³ change	**0.90**	0.83-0.98	**0.85**	0.74-0.97	**0.87**	0.73-1.04	**1.25**	1.03-1.50
**L_{night}**	Never	60-dB vs. <55dB	**1.16**	1.03-1.32	**1.06**	0.84-1.33	**0.83**	0.69-1.01	**1.14**	0.92-1.41
	Current	60-dB vs. <55dB	**0.84**	0.72-1.00	**0.87**	0.70-1.07	**0.99**	0.81-1.22	**0.98**	0.69-1.41
			**Hypertension**		**Atrial fibrillation**		**COPD**		**Pneumonia**	
**NO_X**	Never	20µg/m³ change	**1.05**	0.97-1.13	**0.97**	0.91-1.03	**0.98**	0.79-1.21	**1.09**	0.99-1.20
	Current	20µg/m³ change	**1.07**	0.97-1.18	**0.96**	0.86-1.07	**1.01**	0.87-1.16	**1.07**	0.97-1.18
**$PM_{2.5}$ Traffic**	Never	1µg/m³ change	**1.03**	0.92-1.16	**0.95**	0.86-1.05	**0.98**	0.74-1.32	**1.08**	0.95-1.24
	Current	1µg/m³ change	**1.05**	0.91-1.21	**0.97**	0.83-1.14	**1.01**	0.84-1.22	**1.03**	0.91-1.17
**L_{night}**	Never	60-dB vs. <55dB	**0.99**	0.92-1.06	**0.94**	0.82-1.08	**0.94**	0.72-1.22	**1.09**	0.91-1.31
	Current	60-dB vs. <55dB	**0.98**	0.90-1.08	**0.92**	0.74-1.14	**1.04**	0.91-1.17	**0.90**	0.73-1.11

All Hazard Ratios adjusted for Age, gender, smoking, BMI & IMD.

Table S5 – Hazard ratios for all outcomes during 2005-2011 for pollutants not in main paper - NO_2, $PM_{2.5}$ (exhaust) and $PM_{2.5}$ (traffic non-exhaust)

Exposure	Unit	CHD		MI		Stroke		Heart failure	
		**HR**	95% CI	**HR**	95% CI	**HR**	95% CI	**HR**	95% CI
**NO_2**	10μg/m^3 change	**0.97**	0.91-1.02	**0.88**	0.79-0.97	**0.88**	0.82-0.95	**1.15**	1.02-1.30
**$PM_{2.5}$ Traffic Exhaust**	0.5μg/m^3 change	**0.96**	0.91-1.01	**0.89**	0.82-0.98	**0.90**	0.83-0.97	**1.14**	1.02-1.27
**$PM_{2.5}$ Traffic Non-Exhaust**	0.5μg/m^3 change	**0.97**	0.91-1.04	**0.87**	0.77-0.98	**0.87**	0.79-0.97	**1.16**	1.02-1.31
		**Hypertension**		**Atrial fibrillation**		**COPD**		**Pneumonia**	
**NO_2**	10μg/m^3 change	**1.07**	0.96-1.20	**0.98**	0.91-1.05	**0.98**	0.82-1.18	**1.08**	0.98-1.20
**$PM_{2.5}$ Traffic Exhaust**	0.5μg/m^3 change	**1.04**	0.93-1.15	**0.99**	0.92-1.06	**0.99**	0.84-1.18	**1.04**	0.95-1.14
**$PM_{2.5}$ Traffic Non-Exhaust**	0.5μg/m^3 change	**1.02**	0.90-1.16	**0.98**	0.90-1.06	**0.96**	0.79-1.16	**1.05**	0.95-1.16

All Hazard Ratios adjusted for Age, gender, smoking, BMI & IMD.

Table S6 – Hazard ratios for between and within practice effects for all outcomes during 2005-2011 by NO_x, $PM_{2.5}$ (traffic sources only) and L_{night}

Exposure	Unit	Within or Between practice	CHD HR	CHD 95% CI	MI HR	MI 95% CI	Stroke HR	Stroke 95% CI	Heart failure HR	Heart failure 95% CI
**NO_X**	20µg/m³ change	Between	**0.97**	0.88-1.07	**0.85**	0.73-0.99	**1.02**	0.91-1.15	**1.09**	0.93-1.28
		Within	**1.00**	0.92-1.09	**1.03**	0.91-1.18	**0.89**	0.79-1.00	**1.03**	0.90-1.18
**$PM_{2.5}$ Traffic**	1µg/m³ change	Between	**0.95**	0.84-1.07	**0.79**	0.66-0.95	**0.97**	0.85-1.11	**1.18**	0.96-1.44
		Within	**0.99**	0.91-1.09	**1.02**	0.89-1.18	**0.90**	0.79-1.03	**1.04**	0.90-1.21
**L_{night}**	5 dB change	Between	**0.99**	0.82-1.19	**0.84**	0.63-1.13	**1.06**	0.83-1.35	**1.48**	1.07-2.05
		Within	**0.99**	0.96-1.01	**1.00**	0.96-1.05	**0.97**	0.93-1.01	**1.01**	0.96-1.06

Exposure	Unit	Within or Between practice	Hypertension HR	Hypertension 95% CI	Atrial fibrillation HR	Atrial fibrillation 95% CI	COPD HR	COPD 95% CI	Pneumonia HR	Pneumonia 95% CI
**NO_X**	20µg/m³ change	Between	**1.15**	1.03-1.28	**1.00**	0.89-1.13	**1.06**	0.84-1.33	**1.13**	0.99-1.29
		Within	**0.94**	0.89-0.99	**0.97**	0.88-1.09	**0.94**	0.86-1.03	**0.96**	0.88-1.06
**$PM_{2.5}$ Traffic**	1µg/m³ change	Between	**1.18**	0.99-1.41	**0.99**	0.86-1.13	**1.08**	0.78-1.50	**1.17**	0.98-1.39
		Within	**0.93**	0.87-0.98	**0.99**	0.88-1.11	**0.93**	0.84-1.02	**0.95**	0.85-1.05
**L_{night}**	5 dB change	Between	**1.20**	0.83-1.74	**1.13**	0.92-1.39	**1.15**	0.71-1.87	**1.22**	0.92-1.62
		Within	**0.99**	0.97-1.00	**0.98**	0.96-1.01	**0.98**	0.95-1.01	**0.98**	0.94-1.01

All Hazard Ratios adjusted for Age, gender, smoking, BMI & IMD.
